# Personal

**Published:** 1897-06

**Authors:** 


### Personal.

Dr. Roswell Park, professor of surgery at the Buffalo University Medical College, has been ill for some time, suffering from an infection wound received during an operation that he made about a year and a half ago. He recovered promptly from the initial attack and the infection has remained latent until about four weeks ago. Since that time, however, it has reappeared and on Thursday, May 27, 1897, after deliberate observation and consultation, Dr. John Parmenter, adjunct professor of clinical surgery, assisted by Dr. Chauncey P. Smith, removed a group of infected glands in Dr. Park's left arm. It is believed that this will terminate the manifestation of the malady and that Dr. Park will soon be well and at his surgical work as usual—a hope that his many numerous friends confidently cherish.

Dr. E. C. W. O'Brien, surgeon of the Buffalo fire department, has been appointed consulting surgeon to the Riverside Hospital.

Dr. Joseph M. Mathews, of Louisville, has been elected president of the medical society of the state of Kentucky. In this selection this famous medical society has done itself credit. The next meeting of this society will be held at Maysville in May, 1898, and it will be looked forward to with interest by the profession of the blue grass state.

Dr. Ray H. Johnson, of Buffalo, has removed from 180 North Division street to the corner of North Division and Chestnut streets. Hours: 8–9 a. m., 1.30–2.30 and 7 p. m. Sundays: 1.30–2.30 p. m. Telephone, Seneca 212.

Dr. F. Park Lewis, of Buffalo, has removed from 188 Franklin street to 454 Franklin street, where he will continue the practice of ophthalmology and otology. Hours: 9–1. Examinations by appointment.

Dr. Daniel Lewis, of New York City, has been reëlected president of the State Board of Health. This is a just recognition of efficient services rendered and the Journal is pleased to extend its congratulations to Dr. Lewis.

---

Dr. L. A. Weigel, of Rochester, has removed from 43 Mortimer street to 209 East avenue, where he will continue the practice of orthopedic surgery. Hours: 10–1, except on Wednesday.

---

Dr. Elmer Starr, of Buffalo, has removed from 412 Franklin street to 523 Delaware avenue, where he will continue the practice of ophthalmology. Hours: 9 a. m.—1 p. m.

---

Dr. James A. Gibson, of Buffalo, has removed from 306 Niagara street to 492 Elmwood avenue. Hours: 8–9 a. m., 1.30–2.30 and 7 p. m. Telephone, Bryant 726.

---

Dr. J. J. Kane, of Buffalo, has removed from 322 to 353 Elk street.

